# A Rare Case of Intra-Oral Dirofilariasis Manifesting on The Buccal Mucosa

**DOI:** 10.30699/IJP.2022.548111.2829

**Published:** 2022-08-15

**Authors:** Rakesh Suresh, Mahija Janardhanan, Vindhya Savithri, Thara Aravind

**Affiliations:** Department of Oral & Maxillofacial Pathology and Microbiology, Amrita School of Dentistry, Amrita Vishwa Vidyapeetham, Kochi, India

**Keywords:** Dirofilariasis, Dirofilaria repens, Oral, Sub-cutaneous nodule

## Abstract

Dirofilariasis is a rare zoonotic disease endemic in tropical and sub-tropic countries, including India. Caused by the nematode of the genus Dirofilaria, the disease usually affects canines which form the primary hosts. Humans rarely get infected through the bite of potential mosquito vectors. Manifestations in humans have been reported to affect the orbital region, and intra-oral involvement is rarely reported. Our case was a 5-year-old boy who presented with a slow-growing diffuse swelling on the buccal mucosa. Dirofilariasis was diagnosed when the excised specimen was subject for histopathologic evaluation, yielding the identification of the Dirofilaria worm with the typical morphologic characteristics in the tissue sections. An extremely rare occurrence intra-orally, dirofilariasis can manifest as subcutaneous nodules. Pathologists have an important role in the final diagnosis of the disease through identifying the adult worm in the tissue sections of the biopsy specimen. Dental practitioners must be aware of such an entity as rarely this can be encountered in routine dental practice.

## Introduction

Dirofilariasis is a parasitic infection caused by the helminthic nematode that belongs to the genus *Dirofilaria *and has been reported to occur worldwide. Dirofilaria worms are habitual parasites in canines and can occasionally cause accidental transmission leading to infections in humans. Among the forty recognized species of Dirofilaria, only six are reported to cause disease in man (1). *Dirofilaria repens* and *Dirofilaria tenuis* have been reported to cause sub-cutaneous infection, while *Dirofilaria immitis* is said to produce pulmonary dirofilariasis in humans (2). The species responsible for human dirofilariasis differs with the geographic location. Most human infections in Asia have been reported to be caused by *D. repens*. In India, the few reported cases of human dirofilariasis are from the southern state of Kerala, which points towards a possible focus of infection with *D. repens* in this geographic area (3-5). Dirofilariasis is a vector borne zoonosis and transmission to man occurs through the bite of mosquito vectors. Density of potential mosquito vectors with an extended breeding season, warm climate and abundance of microfilaremic canines may be significant factors in determining the density of human dirofilarial infections in a region (6). The exposed parts of human body which include skin of the head and neck region and the lower extremities constitute the major sub cutaneous sites of involvement. Of these, most of the cases have been reported to affect the ocular and peri-ocular regions (7). Infections involving oral tissues are extremely rare, with only a few cases documented. Dirofilariasis is diagnosed by microscopic evaluation of the excised tissue from the affected area, which yields the identification of the Dirofilaria worms in the tissue sections by their specific morphologic features (8). This article reports a rare finding of human dirofilariasis in the buccal mucosa and the role of histopathologic examination in making an accurate and timely diagnosis for successful management of the entity.

## Case Presentation

**Fig. 1:**
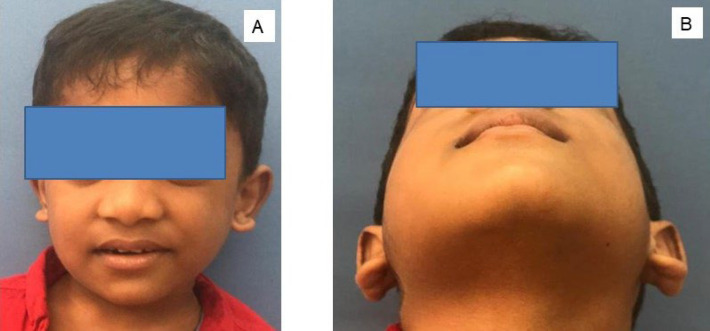
Extra-oral clinical photograph. A) Slight facial asymmetry visible on the right side of the face in the cheek region, B) A diffuse swelling on the right side of the face above the lower border of the mandible in the cheek region

A 5-year-old boy presented with a complaint of a slow-growing swelling of two months on the right side of the face. History revealed that he was treated with antibiotics at a medical clinic, but the swelling did not subside. Extra-orally, there was mild facial asymmetry, and fullness on the lower part of the right cheek that was visible on external examination of the face (Figure 1, A and B). There was no tenderness on palpation of the affected side, but on attempting the intra-oral examination, it was observed that the patient's ability to open his mouth was restricted. The child was non-cooperative during the intra-oral examination, citing discomfort while trying to open the mouth. With the minimal mouth opening possible, the intra-oral site of the swelling was located as the soft tissue in the right buccal vestibule, with the mucosa over the diffuse swelling appearing normal. Palpation of the intra-oral site was also attempted, and the swelling was found to vary from firm to hard in consistency. Orthopantogram revealed no changes in the bone of the mandible corresponding to the site of the swelling; the other radiographic findings were not significant in this case. Fine needle aspiration cytology from the lesion yielded minimal blood-tinged fluid material, and the microscopic examinations revealed only red blood cells. A complete hematologic evaluation of the patient revealed the eosinophil count at 10.6%, suggestive of eosinophilia.

**Fig. 2:**
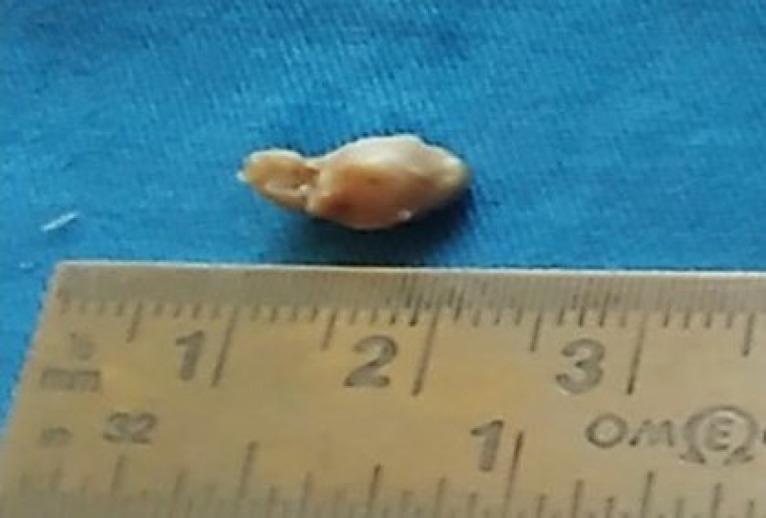
Photograph of the gross specimen. The excised specimen appeared as a brownish white cystic sac with a smooth outer surface

**Fig. 3:**
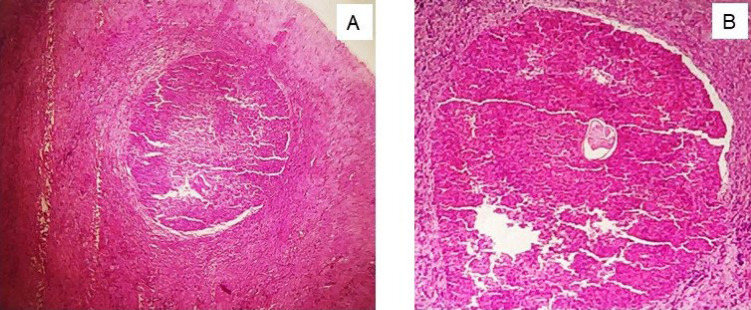
Histologic examination of the excised lesion. A) An intense granulomatous inflammatory response surrounding the parasite (H&E stain, 4X), B) a Cross-section of the adult worm surrounded by a dense inflammatory response (H&E stain, 10X)

**Fig. 4:**
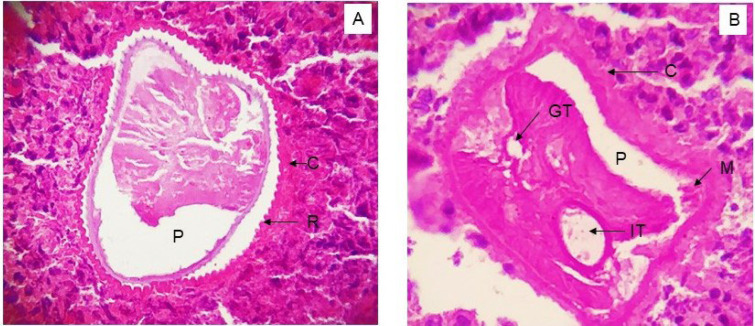
Histologic examination of the excised lesion. A) Cross-sectional morphology of the Dirofilaria worm where the outer cuticle (C) shows longitudinal ridges (R) and an inner pseudocoel cavity (P) (H&E stain, 40X). B) Cross-sectional morphology of the Dirofilaria worm showing a well-developed inner musculature (M) attached to the outer cuticle (C) and lining the peudocoel cavity (P). The intestinal tubule (IT) and the genital tubule (GT) containing spermatocytes are also observed. (H&E stain, 40X)

**Fig. 5:**
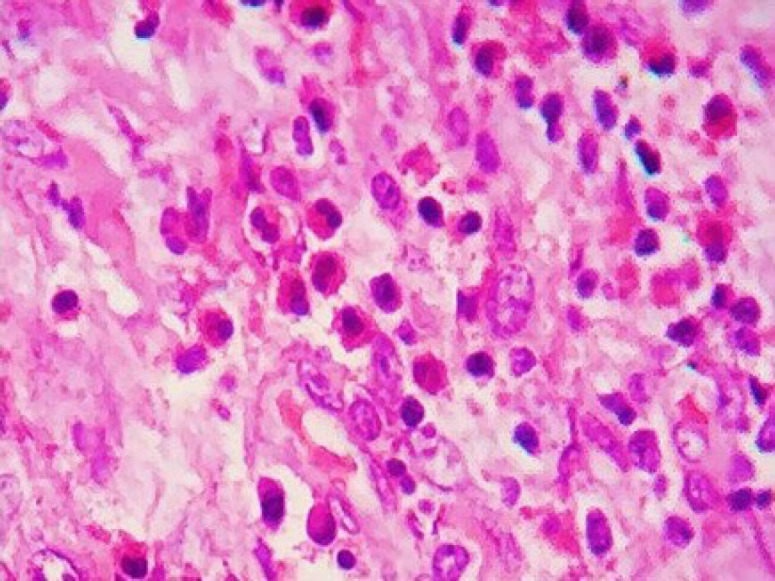
Histologic examination of the excised lesion. Inflammatory cells such as lymphocytes, plasma cells, macrophages, and a few eosinophils were noticed in the inflammatory response surrounding the parasite in the tissue. (H&E stain, 40X)

Excision of the lesion was performed under general anesthesia, and the excised soft tissue specimen was submitted for histopathologic examination. The gross specimen received appeared as a brownish white sac, firm in consistency with thick walls and a smooth outer surface (Figure 2). There was no content within, and representative bits were sectioned and given for processing for histopathologic evaluation. Microscopic examination revealed a cross-section of a single worm surrounded by a dense granulomatous response (Figure 3, A and B). Higher magnification showed the morphologic characteristics of adult filarial worms with outer cuticle (C) showing prominent longitudinal ridges (R) and a well-developed muscular layer (M) lining the pseudocoel cavity (P). Within this cavity, the intestinal tubule (IT) as well as the male genital tubule (GT) containing spermatocytes were seen (Figure 4, A and B). The surrounding granulomatous tissue showed a dense inflammatory cell infiltrate comprising of chiefly lymphocytes and macrophages, along with sprinkling of eosinophils and plasma cells (Figure 5). The morphologic characteristics were consistent with adult dirofilarial worm and hence the lesion was diagnosed as dirofilariasis. The cross-sectional features of the worm in the sections showed a prominent cuticle with longitudinal ridges, each separated from the next by a distance greater than its width and a well-developed musculature on the inner aspect, which were characteristic of D. repens. Based on these features and given the epidemiologic data suggestive of endemic nature of this species in this geographic location, the worm was identified as Dirofilariasis repens. 

Considering the facts that the Surgeons confirmed the more superficial juxta-epithelial location of the lesion in the buccal mucosa as observed during excision and the presence of the cross-sectional features of the worm in the connective tissue below the superficial stratified squamous epithelium of the buccal mucosa as seen in the histologic sections, the intra-mucosal location of the lesion in the buccal mucosa was confirmed. After three weeks, a review of the patient showed complete resolution of the lesion and a satisfactory healing phase.

## Discussion

Dirofilariasis is a parasitic infection of animals that are rarely encountered in humans. The causative organism is the nematode of the genus Dirofilaria (6). This filarial worm is found to be a natural parasite of canines which are the main primary hosts. Most of these worms reside in the infected hosts' subcutaneous tissue, producing microfilariae larvae that circulate in the host's circulation and are transmitted by mosquito vectors of the genre Ades, Anophelex, or Culex (9). The development of the parasite in the mosquito takes about 2 weeks. Man is a rare accidental host where the disease is acquired by the bite of the mosquito vector carrying the microfilariae larvae. If not rapidly destroyed by the host immune response in humans, the parasite larvae gradually grow into adult worms in about 6 months (10). Humans are considered as "dead ends" in the life cycle of Dirofilaria since their further development cannot take place in the human body. Two types of diseases, namely, the sub-cutaneous form usually produced by *D. repens* and *D. tenuis* and the pulmonary form caused by *D. immitis,* have been reported in humans. Most of the reported human cases are of the sub-cutaneous disease, where the worms may produce a granulomatous response leading to a clinical nodule in the subcutaneous tissues (11). Most of the reported human infections show involvement of the eyes, eyelids, and conjunctiva. However, intra-oral involvement in human subjects is infrequent, with just over a hundred cases reported worldwide in literature. Of these, only about 20 cases have been reported from India. A study of these reported intra-oral cases shows a preference for the buccal mucosa, followed by the lips (12, 13). 

The epidemiological data on the disease in India shows that *D. repens* was responsible for most of the reported human infections. The maximum number of cases were reported in the southern state of Kerala (13). Factors such as a high density of appropriate mosquito vectors, favorable climate for vector breeding, and plenty of microfilaremia canines may contribute to the focus of Dirofilaria infection in this specific geographic location (14, 15). 

Biopsy from the affected site and demonstration of the adult Dirofilaria worm in the tissue sections remains the gold standard for diagnosing dirofilariasis. It is important, therefore, for the Pathologists to be familiar with the microscopic characteristics of the Dirofilaria worm in routine tissue sections. A thick cuticle with longitudinal ridges and a well-developed circumferential musculature with two lateral chords are characteristic of Dirofilaria (16). The distinctly rounded striations in the cuticle are characteristic of *D. repens*, where the ridges are separated by a distance wider than the ridge itself. On the other hand, *D. immitis* shows a relatively smooth cuticle without ridges (17). Species identification of the Dirofilaria worm may not always be possible through histopathologic examination since the intense inflammatory response elicited by the parasite in the host tissue may alter or mask the actual morphologic characteristics of the worm in the tissue sections (18). However, in our case, the typical morphologic features of the Dirofilaria worm were clearly visible in the microscopic examination of the patient tissue sections, despite a dense granulomatous reaction surrounding the worm. This made the final diagnosis possible in this case. Though DNA-based molecular techniques can be used for species identification of Dirofilaria, the formalin-fixed biopsy specimens are usually subject to histopathologic evaluation since dirofilariasis may not be considered as a possible clinical diagnosis in most instances. These rule out the possibility of using further molecular techniques for species characterization (14). 

Although dogs are thought to represent the most important reservoirs for this parasite, it has been emphasized recently that the mosquito population is more important for the emergence of human cases (19). With many of the dirofilariasis cases reported in India being from the Southern state of Kerala, the place is thought to be a favorable ground for the potential mosquito vectors of this disease. Our patient possibly got infected through the bite of a mosquito vector that transmitted the Dirofilaria larvae into the patient's circulation. The submucosal nodule in the buccal mucosa of our patient was probably due to the migration of the worm from the facial subcutaneous tissue into the buccal mucosa. Human dirofilariasis is generally regarded as infection by a single worm, and surgical removal of the lesion containing the parasite is adequate to treat the disease. No further treatment is required (20). 

## Conclusion

We have reported a rare case of dirofilariasis involving the buccal mucosa in a five-year-old boy. Dirofilariasis, a zoonotic infection of animals, rarely transmits to humans through the bite of a mosquito vector. Most reported cases of human infections are in the orbital region. Subcutaneous nodule in the buccal mucosa is the most commonly reported manifestation in intra-oral cases. The final diagnosis of dirofilariasis can be confirmed only through demonstration and identification of the adult Dirofilaria worm in the histopathologic sections of the patient's tissue. This emphasizes the importance of pathologists who need to be familiar with the characteristic morphology of the Dirofilaria parasite in the tissue sections to render a timely and accurate diagnosis. Dental practitioners also need to be aware of the possibility of encountering facial and intra-oral sub cutaneous nodules as a clinical manifestation of dirofilariasis, especially in those parts of the world where the potential mosquito vector population is high. Being caused by a single worm, no further treatment is required after surgical removal of the lesion and confirmation of the diagnosis through proper microscopic evaluation of the tissue sections.

## Conflict of Interest

None declared.

## Funding

None.
